# Investigation of the Correlation between Androgen Receptor and ZEB1 and its Value in Progression of Gastric Cancer

**Published:** 2020

**Authors:** Shahrzad Soleymani Fard, Masoud Sotoudeh, Kioomars Saliminejad, Mansour Yazdanbod, Habibollah Mahmoodzadeh, Shaghayegh Kouchaki, Marjan Yaghmaie, Seyed Asadollah Mousavi, Reza Malekzadeh, Kamran Alimoghaddam, Seyed Hamidollah Ghaffari

**Affiliations:** 1. Hematology, Oncology and Stem Cell Transplantation Research Institute, Faculty of Medicine, Tehran University of Medical Sciences, Tehran, Iran; 2. Digestive Oncology Research Center, Digestive Diseases Research Institute, Tehran University of Medical Sciences, Tehran, Iran; 3. Reproductive Biotechnology Research Center, Avicenna Research Institute (ACECR), Tehran, Iran; 4. Department of Surgery, Madaen Hospital, Tehran, Iran; 5. Department of Surgical Oncology, Cancer Institute, Imam Khomeini Hospital Complex, Tehran University of Medical Sciences, Tehran, Iran

**Keywords:** Androgen receptor, Enzalutamide, Gastric cancer, Prognostic marker, Targeted therapy, ZEB1

## Abstract

**Background:**

Zinc-finger Enhancer Binding protein (ZEB1) acts as a transcription factor to promote cancer progression through regulating Epithelial to Mesenchymal Transition (EMT). It is well-known that ZEB1 mRNA expression is directly induced by both Estrogen Receptor (ER) and Progesterone Receptor (PR). Moreover, Androgen Receptor (AR) and PR could bind to the same regulatory element. Since it has been shown that AR overexpresses in Gastric Cancer (GC) as a male-predominant tumor, the goal of this study was to evaluate whether AR could regulate ZEB1 expression in GC.

**Methods:**

The expression profile of ZEB1 in 60 fresh GC and adjacent non-tumor tissues and 50 normal gastric specimens was assessed by qRT-PCR, and the association of ZEB1 expression with clinicopathological features was investigated. Furthermore, possible correlation between ZEB1 and AR was evaluated to elucidate a novel prognostic marker using Kaplan-Meier method and Cox regression model. Finally, molecular interaction of ZEB1 and AR was assessed using a potent AR antagonist in GC cells.

**Results:**

Among GC patients, 70.2% (40/57) overexpressed ZEB1 and 64.91% (37/57) overexpressed AR relative to normal gastric tissues. ZEB1 overexpression was significantly correlated with the AR overexpression in GC patients. Moreover, ZEB1 overexpression was remarkably associated with lower overall survival; however, it was not an independent prognostic factor. Evidence shows that simultaneous evaluation of ZEB1 and AR expression could independently predict survival of GC patients (HR= 2.193, p=0.047).

**Conclusion:**

These findings have clinical importance suggesting simultaneous evaluation of ZEB1 and AR expression as a potential prognostic marker. Moreover, AR may regulate ZEB1 expression in GC cells proposing a possible promising targeted therapy for GC patients.

## Introduction

Although the incidence of Gastric Cancer (GC) shows a decreasing trend in the last decade, most GC patients are initially diagnosed at late TNM (Tumor, Node, Metastasis) stages or with distant metastases who have poor prognosis 1. Therefore, there is a crucial need to introduce proper treatment strategies which could improve the survival of these Advanced Gastric Cancer (AGC) patients. In contrast to new chemotherapy protocols for GC, the 5-years survival rate for AGC patients is about 5 to 20% with a median Overall Survival (OS) of about 12 months 2. Regarding this fact, numerous studies have been devoted to find pivotal molecular pathways to target specific inhibitors. These targeted therapies can be applied alone or in combination with standard chemotherapies. Moreover, the identification of proper markers that precisely predict aggressiveness of GC is urgently warranted. Therefore, the molecular factors responsible for aggressiveness of GC should be assessed profoundly.

It is well-known that EMT has a critical role in the progression and aggressiveness of GC 3. There are numerous studies which investigated the genes essential to the EMT process 3–5.

Zinc finger E-box Binding homeobox 1 (ZEB1) is one of these genes which has been shown to promote metastasis and develop invasion in various cancer types including breast, prostate, colorectal, ovarian and gastric tumors 6–10. Previously, Murai *et al* indicated that GC patients with ZEB1 overexpression had significantly poorer survival than those with ZEB1 underexpression 11. In a recent study, ZEB1 rs431073 polymorphism has been demonstrated as a prognostic marker of GC survival 10. Moreover, in 2019, Xue *et al* revealed that ZEB1 regulates proliferation and EMT of GC *via* modulating Wnt5a and related mechanisms 12.

A member of the evolutionarily conserved nuclear receptor superfamily, androgen receptor is a transcription factor which regulates the expression of several genes 13. It is indicated that Androgen Receptor (AR) could act as an oncoprotein and modulate metastasis and progression of several cancer types 14–16. Recently, some studies have been devoted to assessment of the role of AR in GC as a male-predominant tumor 17,18. They showed that AR has a pivotal role in progression of GC through interacting with EMT-related genes such as E-cadherin.

Besides, some studies have investigated the interaction between ZEB1 and AR in breast and prostate cancer 19–21. Therefore, an attempt was made to investigate any interaction between these two EMT-related genes in GC.

The aim of this study was assessing the ZEB1 expression in GC and normal gastric tissues, its association with clinicopathological characteristics and the potential correlation between *ZEB1* and *AR* genes expression in GC patients. Finally, using an AR antagonist in GC cell lines, the possible interaction between ZEB1 and AR signaling pathways was evaluated aiming to introduce a novel promising therapeutic agent for AGC patients.

## Materials and Methods

### Patients and clinicopathological data

In the present cohort study, 60 fresh tissue samples were collected from GC patients who underwent surgical resection at Madaen, Kasra or Imam Khomeini Hospitals, Tehran, Iran, between June 2016 and June 2017. All patients were pathologically and clinically diagnosed with GC; moreover, patients who received chemotherapy or radiotherapy before surgery or patients with double primary tumors were excluded. Fresh tumor tissue specimens and adjacent non-tumor tissues were prepared within 15 *min* of excision, stabilized in RNA later solution (RNA later RNA stabilization Reagent, QIAGEN, Germany) at 4*°C* overnight and preserved at −20*°C* until RNA extraction. The patients were followed up until death or the end of the study (Sept. 2018). OS refers to the time (months) between the date of surgery and the date of death or at the end of follow-up.

Furthermore, 50 fresh samples were obtained from normal cases who underwent endoscopy procedure in Digestive Diseases Research Institute, Shariati hospital, Tehran, Iran.

The informed consents were signed by all participating patients or their first family members. The Clinical Research Ethics Committee of Tehran University of Medical School (TUMS) has approved our research. This study complies with the ethical principles of the HORC-SCT, Shariati Hospital and the Helsinki Declaration of 1964 and later versions. Ethics committee approval code: ir.TUMS.horcsct.rec.1394.103.10.

### Human GC cell lines

Human GC cell lines were acquired from National Cell Bank of Iran (NCBI; Tehran, Iran). These cells included KATO III, NCBI Code: C640 and MKN45, and NCBI Code: C615. Authentication of these cell lines was carried out by STR (Short Tandem Repeat) profiling using Cell IDTM system (Promega). The cell lines were obtained in July 2017 and all *in vitro* experiments were accomplished fewer than 6 months after receipt. Moreover, cellular morphology was periodically checked. GC cell lines were cultured in RPMI 1640 containing 10% FBS and maintained at 37*°C* under humidified atmosphere with 5% CO_2_.

### Chemicals

Enzalutamide (MDV3100) (An androgen-receptor antagonist with greater affinity to AR than Bicalutamide) was purchased from Selleck chem (Houston, TX, USA) and was dissolved in DMSO. The final concentrations of DMSO did not exceed 0.1% (*v/v*) in all the treatments. MTT [3-(4,5-dimethylthiazol-2-yl)-5-(3-car boxymethoxyphenyl-2-(4-sulfo-phenyl)-2H-tetrazolium] was purchased from SIGMA-ALDRICHs, Steibeim, Germany. RNA later (RNA Stabilization Reagent) was obtained from QIAGEN, Germany.

### Cytotoxicity assays

KATO III and MKN45 cells in logarithmic growth phase were plated at a density of 2500 cells per well in 96-well plates. The cells were exposed to different concentrations of ENZ (0.1–50 *μM*). The viability was assessed 24, 48 and 72 *hr* post treatment by MTT assay. Vehicle-treated cells were used as the control group.

### Total RNA preparation and reverse transcription-PCR

Total RNA was extracted from the RNA later-stabilized tissues or cell line lysates using 1 *ml* RiboEx reagent (Gene All Biotechnology Co, South Korea).

cDNA was synthesized using PrimeScriptTM RT reagent Kit (TaKaRa, Japan). The reaction vessel was incubated in an ABI Veriti Thermocycler (Applied Biosystems). The control gene used in this study was human Beta-2-microglobulin (B2M). RT-PCR was performed with ABI Veriti Thermocycler (Applied Biosystems) using Taq DNA polymerase master mix red (Ampliqon, Copenhagen, Denmark). All PCR products were visualized by 1% agarose gel electrophoresis.GelQuant.NET was used to investigate the intensity of each band.

### Real-time quantitative PCR

A Light Cycler 96 instrument (Roche Molecular Diagnostics) was applied to perform the quantitative reverse transcription-PCR (qRT-PCR) analysis using SYBR Green Real Q-PCR Master Mix kit (Ampliqon, Copenhagen, Denmark) as described by the manufacturer. Thermal cycling condition consisted of an activation step for 15 *min* at 95*°C* followed by 40 cycles of denaturation step (15 *s* at 95*°C*) and a combined annealing/extension step for 1 *min* at 60*°C*. Water instead of cDNA was included in the PCR reaction as negative controls. In the present study, two different housekeeping genes [hypoxanthine phosphoribosyl transferase1 (HPRT) and B2M)] were used for normalization of target genes expression levels. However, B2M proved to be the more stable one among the evaluated genes, and showed no variation between tissues.

mRNA expression levels were quantified as ΔCt values by comparing it with the mean Ct values of B2M taken as reference/endogenous control gene (ΔCt=Ct target-Ct reference) to normalize the possible differences in the amount of total RNA. The relative expression levels were calculated using the 2−(ΔΔCT) method according to the following formula: ΔΔCT= ΔCt tumor–ΔCt normal 22.

In the present study, one sample from normal cases which had the highest ΔCt value was used as a calibrator for each specific gene. All other samples from three different groups (tumor tissues, non-tumor adjacent tissues and normal tissues) were compared with the calibrator to calculate the fold change in gene expression. Next, the cut off value was determined using Receiver Operating Characteristic curve (ROC curve) for all mentioned genes. Values higher than cut off point were considered as overexpression and the values lower than cut off point were considered as under expression. The sequences of primers used in the present study are listed in table 1.

**Table 1.** Nucleotide sequences of the primers used for QRT-PCR

**Gene**	**Accession number**	**Forward Primer**	**Reverse Primer**
**B2M**	NM_004048	GATGAGTATGCCTGCCGTGT	CTGCTTACATGTCTCGATCCCA
**HPRT**	NM_000194	TGGACAGGACTGAACGTCTTG	CCAGCAGGTCAGCAAAGAATTTA
**AR**	NM_000044	TTGTCCATCTTGTCGTCTTCGG	GCCTCTCCTTCCTCCTGTAGT
**ZEB1**	XM_017016597.1	CTACAACAACAAGACACTGCTGT	TGTTCTTTCAGAGAGGTAAACCG

### Statistical analysis

Difference in expression of ZEB1 between gastric tumors and adjacent non-tumor tissues or normal tissues was compared by independent samples Mann-Whitney U test. Correlation was computed using Spearman rank test. The associations between expression of ZEB1 and clinicopathological characteristics were evaluated using Chi-square or Fisher’s exact tests.

The survival rate was analyzed by Kaplan-Meier method (Log-rank test). Univariate and multivariate survival analysis was performed by the Cox proportional hazards model to evaluate the prognostic value of known categorical variables and ZEB1 expression. All significant factors (p<0.05) in the univariate analysis were used for multivariate evaluation. Stepwise backward elimination was used till only significant variables were maintained in multivariate model. Functional experiments analyzed by two-tailed Student’s t-test and the data are presented as the mean ± standard deviation from three independent experiments. Computerized statistical analyses were performed by the IBM SPSS® statistics 22 software. p<0.05 was considered statistically significant.

## Results

### Clinicopathological characteristics

Clinicopathological characteristics of GC patient is listed in table 2. Fifty normal cases including 25 females and 25 males (age median and range 51 and 19–83 years, respectively) were also collected.

**Table 2.** Association between ZEB1 expression and clinicopathological characteristics of patients with gastric cancer

**Clinical variables**	**Total patients: n (%)**	**Evaluable patients: n (%)**		**p**

	**57 (100)**	**Overexpressed No. (%)**	**Underexpressed No. (%)**	
**Age (years) median, range**	63, 33–83			
n <63	28 (49.1)	19 (33.3)	9 (15.8)	0.707
n ≥63	29 (50.9)	21 (36.8)	8 (14)	
**Sex**				
Male	37 (64.9)	24 (42.1)	13 (22.8)	0.233
Female	20 (35.1)	16 (28.1)	4 (7)	
**Tumor size (*cm*)**				
n <5	17 (29.8)	9 (15.8)	8 (14)	**0.054**
n ≥5	40 (70)	31 (54.4)	9 (15.8)	
**Lauren’s classification**				
Intestinal	53 (93)	37 (64.9)	16 (28.1)	0.657
Diffuse	4 (7)	3 (5.3)	1 (1.8)	
**Tumor grade**				
Poorly differentiated	30 (52.6)	22 (38.6)	8 (14)	0.917
Moderately differentiated	21 (36.8)	14 (24.6)	7 (12.3)	
Well differentiated	6 (10.5)	4 (7)	2 (3.5)	
**Tumor type**				
Adenocarcinoma	44 (77.2)	31 (54.4)	13 (22.8)	0.592
Signet ring cell carcinoma	13 (22.8)	9 (15.8)	4 (7)	
**Lymphovascular invasion**				
YES	40 (70.2)	31 (54.4)	9 (15.8)	**0.046**
NO	17 (29.8)	9 (15.8)	8 (14)	
**Perineural invasion**				
YES	47 (82.5)	34 (59.6)	13 (22.8)	0.464
NO	10 (17.5)	6 (10.5)	4 (7)	
**Tumor shape**				
Ulcerated flat	46 (80.7)	32 (56.1)	14 (24.6)	0.537
Linitis plastica	4 (7)	3 (5.3)	1 (1.8)	
Polypoid	7 (12.3)	5 (8.8)	2 (3.5)	
**Tumor location**				
Proximal	26 (45.6)	17 (29.8)	9 (15.8)	0.474
Middle	22 (38.6)	16 (28.1)	6 (10.5)	
Distal	6 (10.5)	4 (7)	2 (3.5)	
Diffuse	3 (5.3)	3 (5.3)	0 (0)	
**T classification**				
pT1	0 (0)	0 (0)	0 (0)	0.610
pT2	12 (21.1)	8 (14)	4 (7)	
pT3	19 (33.3)	12 (21.1)	7 (12.3)	
pT4	26 (45.6)	20 (35.1)	6 (10.5)	
**N classification**				
N0	19 (33.3)	10 (17.5)	9 (15.8)	0.222
N1	10 (17.5)	7 (12.3)	3 (5.3)	
N2	16 (28.1)	13 (22.8)	3 (5.3)	
N3	12 (21.1)	10 (17.5)	2 (3.5)	
**M classification**				
M0	41 (71.9)	28 (49.1)	13 (22.8)	0.438
M1	16 (28.1)	12 (21.1)	4 (7)	
**TNM stage**				
I + II	22 (38.6)	12 (21.1)	10 (17.5)	**0.041**
III + IV	35 (61.4)	28 (49.1)	7 (12.3)	
**AR Expression**				
Underexpressed	20 (35)	7 (12.3)	13 (22.7)	**0.037**
Overexpressed	37 (64.9)	33 (57.9)	4 (7)	

ZEB1, Zinc-finger E-box-binding homeobox factor 1. AR, Androgen Receptor. # The 8th TNM Classification of Malignant Tumors proposed by the AJCC/UICC.

ZEB1 overexpression has been shown to have a statistically significant correlation with lymphovascular invasion, advanced TNM stages and *AR* gene overexpression (Table 2). Among 37 GC patients overexpressing AR, only 4 patients underexpressed ZEB1. Moreover, ZEB1 overexpression was marginally significant when GC tumor size is concerned. 31 patients out of 40 cases who had tumor bigger than 5 *cm*, overexpressed ZEB1 gene (77.5%). No remarkable association was found between ZEB1 expression and age or gender. The analysis is based on comparing tumors tissues which showed increased ZEB1 expression with normal gastric tissues.

### ZEB1 expression in GC and normal tissues

**Figure 1.**
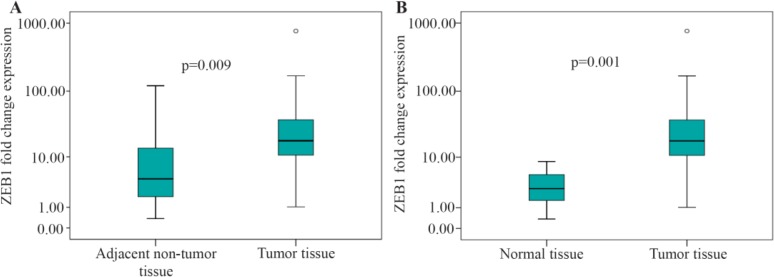
Graphical box-plot expression profile at transcriptome level. Comparing ZEB1 expression in gastric tumor tissues with (A) adjacent non-tumor and (B) normal tissues. Results are the mean of three independent experiments±SD (p<0.05).

Quantitative real-time PCR was used to detect the relative ZEB1 mRNA expression in gastric samples (Figure 1).

The results showed significantly higher values of ZEB1 expression in GC tissues compared to adjacent non-tumor tissues and normal tissues (median of fold change expression, 17.95 *vs.* 4.23, p=0.009; 17.95 *vs.* 2.81, p<0.001, respectively).

### Correlations between mRNA expression of ZEB1 and androgen receptor

**Figure 2.**
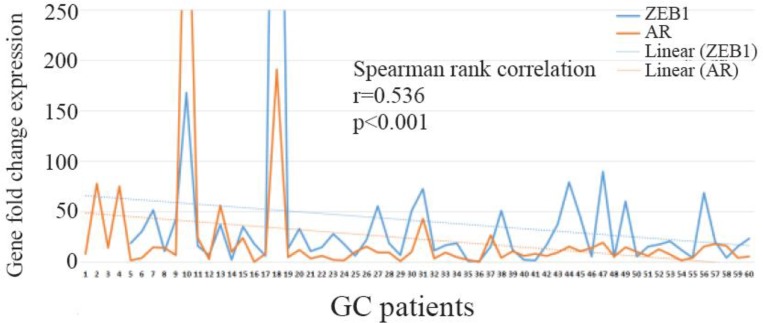
Relationship between AR and ZEB1 expression in GC samples using Spearman rank test.

Spearman rank test was applied to determine the correlation between expression of ZEB1 and AR. efficient was detected between these two genes expression (r=0.536, p<0.001) (Figure 2). Among groups of patients overexpressing ZEB1, higher values of AR mRNA expression were observed.

### ZEB1 expression correlates with OS of GC patients

**Figure 3.**
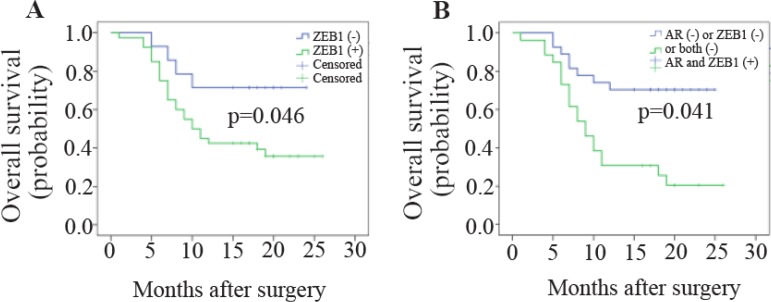
Kaplan-Meier curves of OS for GC patients according to (A) ZEB1 expression, (B) OS for GC patients who simultaneously overexpressed ZEB1 and AR (Log-rank test).

In the present study, GC patients were followed up for 26 months after their surgery. Three patients who failed to contact were lost for follow up. Kaplan-Meier analysis revealed a significant correlation between ZEB1 mRNA expression and OS of GC patients (Figure 3A). Among patients who overexpressed ZEB1, 62.5% and among patients underexpressed ZEB1, 28.6% passed away during this study. Moreover, GC patients who simultaneously overexpressed *ZEB1* and *AR* genes were asked whether they had worse outcome (lower OS) than other GC patients (Figure 3B). Interestingly, survival analysis revealed the higher rate of death among these GC patients (76.9%).

Additionally, univariate and multivariate Cox regression analysis was applied to measure the prognostic role of clinicopathological characteristics and ZEB1 expression in GC patient (Table 3).

**Table 3.** Univariate and multivariate Cox regression analysis of overall survival in patients with gastric cancer

**Variable**	**Univariate cox**			**Multivariate cox**		

	**HR**	**95% CI**	**p**	**HR**	**95% CI**	**p**
**Sex**						
Male	1.000					
Female	0.771	0.378–1.574	0.475			
**Age (years)**	0.999	0.472–1.942	0.903			
**Tumor size (*cm*)**						
n <5	1.000			1.000		
n ≥5	2.588	0.993–6.742	**0.052**	1.547	0.535–4.471	0.421
**Lauren’s classification**						
Intestinal	1.000					
Diffuse	2.388	0.741–7.912	0. 142			
**Tumor grade**						
Well differentiated	1.000					
Moderately differentiated	1.184	0.272–5.150	0.822			
Poorly differentiated	1.337	0.296–6.040	0.705			
**Perineural invasion**						
No	1.000					
Yes	3.019	0.736–12.665	0.181			
**Lymphovascular invasion**						
NO	1.000	1.000				
YES	2.587	0.990–6.759	0.052	1.291	0.411–4.056	0.662
**T classification**						
pT1						
pT2	1.000	1.000				
pT3	2.173	0.576–7.9	0.245	2.457	0.516–11.709	0.259
pT4	3.782	1.085–12.829	**0.033**	1.502	0.315–7.171	0.610
**N classification**						
N0	1.000	1.000				
N1	4.924	1.268–19.116	**0.024**	2.344	0.470–11.687	0.299
N2	7.435	2.080–26.573	**0.002**	2.056	0.363–11.652	0.261
N3	9.801	2.661–36.664	**0.001**	2.714	0.846–15.482	0.128
**M classification**						
M0	1.000	1.000				
M1	3.178	1.566–6.471	**0.001**	2.323	1. 345–4.013	**0.023**
**TNM stage**						
I + II	1.000	1.000				
III + IV	8.119	2.429–26.411	**0.001**	5.690	1.641–19.725	**0.006**
**ZEB1**						
Underexpressed	1.000	1.000				
Overexpressed	2.743	1.009–6.420	**0.048**	1.130	0.256–4.105	0.455
**AR**						
Underexpressed	1.000	1.000				
Overexpressed	4.147	1.582–10.874	**0.004**	2.425	0.898–6.547	0.070
**ZEB1 and AR**						
ZEB1 or AR or both under expressed	1.000	1.000				
Both overexpressed	3.598	1.641–11.116	**0.002**	2.193	1.02–5.136	**0.047**

HR, Hazard Ratio; CI, Confidence Interval. # The 8th TNM Classification of Malignant Tumors proposed by the AJCC/UICC.

It was recently detected that *AR* gene overexpression associates with poor prognosis of GC patients. In the present study, among all clinicopathological characteristics, T classification, N classification, M classification, advanced TNM stages, *ZEB1* gene overexpression and simultaneous overexpression of ZEB1 and AR were significantly correlated with survival of GC patients. Tumor size was marginally significant. However, multivariate analysis showed that after adjustment with other, only M classification, TNM stage and simultaneous overexpression of ZEB1 and AR were independent prognostic factors.

### Alteration in AR signaling affects ZEB1 gene expression

**Figure 4.**
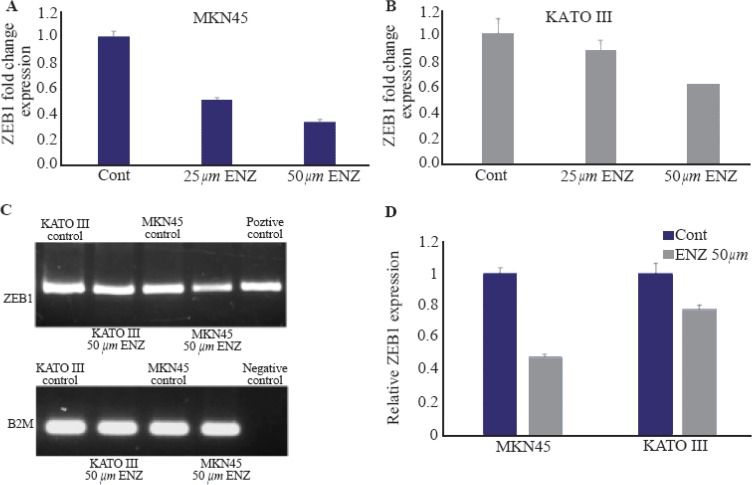
The effects of ENZ on GC cell lines. (A, B). The effect of ENZ on ZEB1 gene expression. After 48 *hr* of treatment with ENZ (25 and 50 *μM*), the GC cells were harvested for quantitative real-time PCR test. Gene expression levels were normalized to B2M. Data are given as mean±SD. Statistically significant values of *p<0.05, **p<0.01, and ***p<0.001 were determined compared with the control. (C) ZEB1 mRNA expression in GC cell detected by RT-PCR after 48 *hr* of treatment with ENZ (50 *μ*). Digital images of the gels were captured by a bio-Rad gel documentation system using Image Lab Software. The samples derived from the same experiment and gels were processed in parallel. DDW was used for negative control and genes expression in LNCaP prostate cancer cells for positive control (D) Analysis of the RT-PCR band intensity using GelQuant. NET.

In the present study, two GC cell lines (MKN45 and KATO III) were used for further assessment of the possible crosstalk between ZEB1 and AR. A novel AR antagonist (Enzalutamide) 23 was applied to assess whether an AR signaling inhibitor could affect ZEB1 mRNA expression in GC cells. The concentrations used mostly in similar studies were up to 50micro molar. Therefore, GC cells were treated with concentrations ranging from 0.1 to 50 *μM*. It was revealed that treatment of GC cells with ENZ for 48 *hr* could significantly decrease ZEB1 mRNA expression in a concentration-dependent manner in both cell lines (Figures 4A and B). However, no significant difference was observed before 48 *hr* in fold change gene expression in any concentration. Moreover, higher concentrations of ENZ (more than 50 *μM*) had killed GC cells in only 24 *hr*, thus there were no cells for extracting their mRNA and assessing ZEB1 expression.

RT-PCR results were found to be in accordance with the quantitative real-time PCR data (Figure 4C). Analyzing the RT-PCR band intensity showed that MKN45 cells were more sensitive to ENZ treatment.

## Discussion

Metastasis, a hallmark of all types of tumors, is the leading cause of approximately 90% of cancer patients’ deaths 3. During EMT process, cancer cells lose adhesion and then expand their motility and aggressiveness dramatically. Various cell signaling pathways are known to trigger the induction and maintenance of EMT such as Wnt/beta-catenin, TGF-beta, E-cadherin, Snail, Twist, STAT3, and ZEB1 3,24.

ZEB1 is a family member of E-box binding transcription factor that contains seven zinc fingers, and can activate or repress its target genes 25. Many studies have reported ZEB1 as a pivotal player in cancer progression by regulating EMT in gastric, breast, prostate, ovarian and colorectal cancers 9,24–27. Moreover, some studies have demonstrated the prognostic role of ZEB1 in GC 24,28.

It is indicated that AR can independently work with EMT-related transcription factors and function as oncogene resulting in aggressive phenotype 21. Recently, the oncogenic role of AR in GC by crosstalk with EMT-related genes such as E-cadherin and β-catenin was demonstrated which was in accordance with some new studies 17,29.

In the present study, the clinical importance of ZEB1 expression and its correlation with AR expression were assessed in GC using tumor and adjacent non-tumor tissues from GC patients, normal gastric tissues from normal cases and two GC cell lines.

It was revealed that *ZEB1* gene overexpression significantly associates with lymphovascular invasion, advanced TNM stages and AR overexpression. This result is consistent with previous study investigating ZEB1 expression in GC 12,24,30. However, Yabusaki *et al* reported that ZEB1 overexpression is only correlated with T classification 26.

Despite several studies evaluating the role of ZEB1 in GC prognosis, their results are controversial. It was demonstrated that *ZEB1* gene expression in GC tissues is higher than adjacent non-tumor or normal gastric tissues. Furthermore, patients who overexpressed ZEB1 had significantly lower OS. Although these results are in concordance with some previous studies 11,26,30, Xue *et al* showed that patients with low ZEB1 expression have lower OS 12.

Also, Cox regression analysis was used to find out whether *ZEB1* gene expression could be used as an independent prognostic factor in GC. It has been disclosed that although ZEB1 overexpression was significantly associated with OS of GC patients (HR=2.743, p=0.048), it cannot be used as an independent prognostic factor after adjustment for other variables entered to the model. Our results are consistent with previous studies 25,26. However, Okugawa *et al* claimed that ZEB1 overexpression is an independent factor for predicting the outcome of GC patients (HR:2.93, p<0.001) 28. Because of these contradictory results, a novel prognostic marker was introduced which could precisely forecast GC patients’ prognosis.

Since the oncogenic role of AR in gastric carcinogenesis by interacting with EMT-related genes has been revealed recently, whether it has crosstalk with ZEB1 as another EMT-related signaling pathway was an issue of interest. The reasoning behind this hypothesis is rooted in the following evidence.

Firstly, many studies have indicated that ZEB1 and AR both interact with some common signaling pathways including E-cadherin, β-catenin, Twist and Snail pathways which are involved in invasion of tumors 31–37. For instance, as revealed recently, a study demonstrated that AR can attach to regulatory sequence of *E-cadherin* gene which results in reducing E-cadherin expression and enhancing metastasis in breast and colon cancer cells 35. Similarly, it has been shown that ZEB1 could attach to *CDH1* gene promoter and reduce the expression of E-cadherin 38.

Secondly, several previous researches have demonstrated the interplay between ZEB1 and AR signaling in various tumor types 9,20,21. Graham *et al* showed that ZEB1 and AR crosstalk promotes metastasis and cell migration in triple negative breast cancer.

In the present study, for the first time, the association of *ZEB1* and *AR* genes expression in GC patients was evaluated. It is revealed that GC patients overexpressing ZEB1 significantly overexpressed *AR* gene at the transcriptional level (p=0.037). Moreover, Spearman rank test demonstrated significant correlation coefficient (r=0.536) between these two genes expression in GC patients.

Additionally, this study provides a clue about the functional interaction between ZEB1 and AR signaling pathways in gastric cancer. We recently indicated the anti-tumor effects of ENZ on the proliferation of GC cells. Here we showed that inhibition of AR signaling using ENZ could alter the mRNA expression of ZEB1 in GC cell lines in a dose-dependent manner. These results are in accordance with previous studies on prostate cancer cells which demonstrated the alteration of ZEB1 expression on mRNA and protein levels 20,21.

Our results have provided the evidence suggesting that simultaneous evaluation of ZEB1 and AR mRNA expression attained a more precise prognostic marker for GC patients’ outcomes. Fascinatingly, simultaneous over-expression of *ZEB1* and *AR* genes, as a single variable, turned out to be independent unfavorable factor for OS of GC patients adjusted for other variables using multivariate Cox regression model (HR=2.193, p= 0.047).

Thus, our data indicate that ZEB1 and androgen receptor signaling pathways have indisputable promising clinical potentials to design novel targeted therapy and use as new prognostic marker for GC patients.

## Conclusion

In summary, this is the first attempt proposing a role for crosstalk between ZEB1 and AR pathways in GC progression and metastasis. Up to our knowledge, no reports have indicated clinical significance associated with regulation of ZEB1 expression by AR in GC. Our study provided evidence explaining a possible encouraging marker, simultaneous assessment of ZEB1 and AR expression, which could appropriately forecast prognosis of GC patients. Moreover, our preliminary results have indicated that ZEB1 mRNA levels could decrease in response to a potent AR antagonist, Enzalutamide, in GC cell lines confirming our observations in GC samples which have shown a significant association between these two genes. However, the use of ZEB1/AR pathways as a prognostic marker and therapeutic target in GC patients warrants further investigation to explore the exact mechanism of interaction between them besides assessment of anti-AR therapy in GC patients *via* larger cohort studies and randomized clinical trials.
